# The *in vitro *effect of gefitinib ('Iressa') alone and in combination with cytotoxic chemotherapy on human solid tumours

**DOI:** 10.1186/1471-2407-4-83

**Published:** 2004-11-23

**Authors:** Louise A Knight, Federica Di Nicolantonio, Pauline Whitehouse, Stuart Mercer, Sanjay Sharma, Sharon Glaysher, Penny Johnson, Ian A Cree

**Affiliations:** 1 Translational Oncology Research Centre, Department of Histopathology, Queen Alexandra Hospital, Portsmouth, UK; 2 Mayday University Hospital, Croydon, UK; 3 Richards Hospital, Chichester, UK

## Abstract

**Background:**

Activation of the epidermal growth factor receptor (EGFR) triggers downstream signaling pathways that regulate many cellular processes involved in tumour survival and growth. Gefitinib ('Iressa') is an orally active tyrosine kinase inhibitor (TKI) targeted to the ATP-binding domain of EGFR (HER1; erbB1).

**Methods:**

In this study we have used a standardised ATP-based tumour chemosensitivity assay (ATP-TCA) to measure the activity of gefitinib alone or in combination with different cytotoxic drugs (cisplatin, gemcitabine, oxaliplatin and treosulfan) against a variety of solid tumours (n = 86), including breast, colorectal, oesophageal and ovarian cancer, carcinoma of unknown primary site, cutaneous and uveal melanoma, non-small cell lung cancer (NSCLC) and sarcoma. The IC50 and IC90 were calculated for each single agent or combination. To allow comparison between samples the Index_SUM _was calculated based on the percentage tumour growth inhibition (TGI) at each test drug concentration (TDC). Gefitinib was tested at concentrations ranging from 0.0625–2 microM (TDC = 0.446 microg/ml). This study represents the first use of a TKI in the assay.

**Results:**

There was heterogeneity in the degree of TGI observed when tumours were tested against single agent gefitinib. 7% (6/86) of tumours exhibited considerable inhibition, but most showed a more modest response resulting in a low TGI. The median IC50 value for single agent gefitinib in all tumours tested was 3.98 microM. Interestingly, gefitinib had both positive and negative effects when used in combination with different cytotoxics. In 59% (45/76) of tumours tested, the addition of gefitinib appeared to potentiate the effect of the cytotoxic agent or combination (of these, 11% (5/45) had a >50% decrease in their Index_SUM_). In 38% of tumours (29/76), the TGI was decreased when the combination of gefitinib + cytotoxic was used in comparison to the cytotoxic alone. In the remaining 3% (2/76) there was no change observed.

**Conclusion:**

The *in vitro *model suggests that gefitinib may have differential effects in response to concomitant cytotoxic chemotherapy with the agents tested during this study. The mechanism involved may relate to the effect of TKIs on growth rate versus their effect on the ability of the cell to survive the stimulus to apoptosis produced by chemotherapy.

## Background

The epidermal growth factor receptor (EGFR) is involved in many cellular processes including cell proliferation, motility, adhesion and angiogenesis via the activation of three pathways: phosphatidylinositol-3 kinase (PI3)/Akt pathway, the Jak/STAT pathway and the ras/raf pathway. EGFR is expressed or highly expressed in a variety of human tumours including non-small-cell lung cancer (NSCLC), breast, bladder, ovarian and head and neck [1] and is therefore a promising target for cancer therapy.

Gefitinib ('Iressa') is an EGFR-tyrosine kinase inhibitor (EGFR-TKI) that competitively inhibits binding of ATP at the ATP site on EGFR. It also displays remarkable selectivity for EGFR (IC50 = 0.033 microM) compared with other receptor tyrosine kinases (RTKs) that share sequence homology in the ATP binding domain [2]. In pre-clinical studies, gefitinib has demonstrated *in vitro *growth inhibition against a variety of human cell lines including NSCLC, ovarian, breast, colon and head and neck and is active in a range of xenograft models, including breast, colon and prostate [3]. Phase II trials with gefitinib monotherapy have produced encouraging results with clinically significant benefits observed, such as disease control rates at 250 mg/day gefitinib of 54% and 42% in IDEAL 1 and IDEAL 2, respectively [4,5]. Results from Phase III trials investigating gefitinib in combination with cisplatin and gemcitabine (INTACT 1) [6] and gefitinib in combination with paclitaxel and carboplatin (INTACT 2) [7] in NSCLC concluded there was no added benefit in patients receiving chemotherapy plus gefitinib; however the tolerability of gefitinib was confirmed.

At present, there is conflicting evidence relating the activity of gefitinib directly to the levels of EGFR expression. One group found that the concentration of gefitinib required to inhibit ligand-independent growth by 50% (IC50) in four bladder cancer cell lines ranged from 1.8–9.7 microM and correlated with EGFR protein and transcript level [8]. However, another study using human tumour xenografts found that gefitinib caused growth inhibition of tumours and enhancement of the activity of a number of cytotoxic drugs, but neither was dependent on high levels of EGFR expression [9]. Moreover, no consistent association was demonstrated between EGFR expression and clinical outcome in IDEAL 1 and 2 [10]. Alternative explanations for the activity of gefitinib in systems where EGFR is not over expressed include inhibition of EGFR pathway activation mediated by increased levels of receptor ligands e.g. epidermal growth factor (EGF) and transforming growth factor-alpha (TGF-alpha); heterodimerization with HER2 and cross talk with heterologous receptors; and EGFR mutations yielding a constitutively active receptor that is not down-regulated by endocytosis [11]. There is evidence that the ras/raf pathway mediates proliferation [12], whereas the PI3/Akt pathway is essential for cell survival and may be constitutively activated in many tumours by loss of PTEN [13].

We have previously shown that the ATP-based tumour chemosensitivity assay (ATP-TCA) can be used to measure the effects of cytotoxic agents and antibodies against human tumour-derived cells, and that this matches clinical outcome in a number of tumour types [14,15]. Use of the assay to direct choice of chemotherapy has been shown to improve response rate and progression-free survival in ovarian cancer [16,17] and a fully randomized trial of assay-directed versus physician's choice of chemotherapy for platinum-resistant ovarian cancer is in progress [18]. The assay system has been used to assist the development of a number of new agents and combinations [19,20], but this represents the first use of a TKI in the assay.

EGF and TGF-alpha, ligands of EGFR, act as survival factors for many cells as well as growth factors. As many cytotoxic agents induce apoptosis, gefitinib may be able to potentiate their effects by reducing survival stimuli. The current pilot study was undertaken to assess the effect of gefitinib in combination with existing chemotherapeutic agents (cisplatin, gemcitabine, oxaliplatin, treosulfan) against a wide range of tumour types.

## Methods

### Tumours

A total of 86 tumours (57 females:29 males) were tested in this study, with a median age of 59 years (range 21–90). The samples tested consisted of the following tumour types; breast adenocarcinoma (n = 8), colorectal carcinoma (n = 18), cutaneous melanoma (n = 7), NSCLC (n = 1), oesophageal adenocarcinoma (n = 4), ovarian carcinoma (n = 26), sarcoma (n = 2), squamous cell carcinoma (n = 2), sweat gland carcinoma (n = 1), uveal melanoma (n = 12) and carcinoma of unknown primary site (n = 5). The 26 ovarian carcinomas were all recurrent stage 3/4 cancers and 25/26 were pre-treated (11 with carboplatin and 14 with carboplatin + paclitaxel). Of the remaining samples, 10/86 had been treated with a variety of chemotherapy regimens and some patients had more than one treatment; epirubicin + cisplatin + 5-Fluorouracil (5-FU) (n = 3), epirubicin + cyclophosphamide (4-HC) (n = 1), 4-HC+methotrexate+5-FU (CMF) (n = 2), cisplatin + vinorelbine (n = 1), mitomycin C + 5-FU (n = 1), mitoxantrone + paclitaxel (n = 1), chlorambucil (n = 1), 4-HC (n = 1) and irinotecan (n = 1). The remaining 51 patients had no previous treatment. In each case only tumour material not required for diagnosis was sent for ATP-TCA, and in all cases consent had been obtained and permission had been granted by the local ethics committee.

### ATP-TCA

The ATP-TCA was performed as previously published [14,21]. Solid tumour or ascites samples were transported to the laboratory in transport medium, consisting of Dulbecco's Eagles Media (DMEM) (Sigma, UK; D6171). Solid samples were dissected under sterile conditions in a BioQ Microfuge Class II Hood and placed into a 0.75 mg/ml collagenase solution (Sigma, UK; C8051) for enzymatic dissociation overnight. Following dissociation, the single celled suspension or ascites sample was washed using DMEM supplemented with 1 M HEPES, (Sigma, UK; H0887), 100 IU/mL penicillin, 10 mg/mL streptomycin (Sigma, UK; P0781) and 10 mg/mL gentamicin (Sigma, UK; G1272). The final cell suspension was then plated in 96-well polypropylene plates (Corning Life Sciences, High Wycombe, UK) at 20,000 (solid sample) or 10,000 (ascites sample) cells/well in a serum-free complete assay medium (CAM, DCS Innovative Diagnostik Systeme, Hamburg, Germany). Drugs were added to triplicate wells at serial dilutions corresponding to 200–6.25% of a test drug concentration (TDC) estimated from pharmacokinetic data, which included the degree of protein binding. Two controls were included in each plate: one with no drug and consisting of media only (MO), and a maximum inhibitor (MI) control which killed all cells present. The plates were incubated for 6 days at 37°C with 5% CO2. At the end of the incubation period, remaining cells were lysed by addition of an ATP extraction reagent (DCS Innovative Diagnostik Systeme). An aliquot of the lysate from each well was added to the corresponding wells of a white 96 well microplate (Thermo Life Sciences, Basingstoke, UK), followed by addition of luciferin-luciferase reagent. The light output corresponding to the level of ATP present was measured in a luminometer (MPLX, Berthold Diagnostic Systems, Hamburg, Germany). These data were transferred automatically to an Excel spreadsheet where the % inhibition achieved at each concentration tested was calculated using the equation; 1-(test-MI)/(MO-MI) × 100. Several parameters of efficacy can be calculated e.g. IC50 and IC90, however previous ATP-TCA studies have found that a natural logarithmic sum index (Index_SUM_) calculated by direct addition of the percentage survival at each concentration tested (Index = 600-Σb3;%Inhibition6.25...200) provides a better indication of sensitivity or resistance to different drugs in different tumour types [22]. The total inhibition of growth resulted in an index of 0, and no inhibition of growth at any concentrations produces an index of 600 [23]. Area under the concentration-inhibition curve (Index_AUC_) was calculated from the data using the trapezoidal rule.

### Data Analysis

The results were entered into an Access 2000 database for further analysis. Statistical tests were performed using non-parametric methods.

### Drugs

The cytotoxic drugs used in the assay were obtained as vials for injection and made up according to manufacturers' instructions. Gemcitabine, oxaliplatin and treosulfan were stored in aliquots at -20°C, while cisplatin was stored at room temperature. Table 1 shows the 100% TDC for each of the drugs used. Drug combinations were tested by combining single agents. The EGFR-TKI, gefitinib (kindly provided by AstraZeneca) was tested at concentrations ranging from 0.06–2 microM (100% TDC = 0.99 microM).

### Immunohistochemistry

Tissue was available for EGFR immunohistochemical staining in 31/86 (36%) cases comprising of 4 breast carcinomas, 12 colon carcinomas, 2 oesophageal carcinomas, 2 ovarian carcinomas, 1 sarcoma, 4 skin melanomas, 5 uveal melanomas and 1 carcinoma of unknown primary site. Paraffin embedded sections of 4 μm thick were dewaxed and rehydrated in preparation for immunohistochemical staining. Endogenous peroxidase was blocked using 3% hydrogen peroxide in methanol. The sections were pretreated with 0.1% Trypsin (CaCl2/Tris buffer pH8.0) for 10 minutes at 37°C for antigen retrieval. Immunohistochemical studies were performed according to manufacturer's instructions of the Vectastain Universal ABC-AP kit (Vector Laboratories, Burlingame, California, U.S.A), which uses an avidin-biotin complex method and Vector red as the chromogen. Monoclonal antibody for EGFR, Clone E30 (Dakocytomation, Cambridgeshire, U.K) was used at a dilution of 1:20 and incubated with sections for 18 hours at 4°C. Positive (squamous cell carcinoma tissue) and negative controls were included in each staining procedure. Samples were assessed by a pathologist using the H-score. Intensity was graded on a scale ranging between 0, 1+, 2+ or 3+, (where 1+ equals weak staining, 2+ equals moderate and 3+ equals intense) and the proportion of cells stained at the highest intensity. The two values were then multiplied together to give the final value.

The same tissue available for EGFR staining was also available for pAkt staining. Paraffin embedded sections of 4 μm thick were dewaxed and rehydrated in preparation for immunohistochemical staining. Endogenous peroxidase was blocked using 3% hydrogen peroxide in methanol. The sections were pretreated with 0.1 M citrate buffer in a pressure cooker for 2.5 minutes for antigen retrieval. Immunohistochemical studies were performed according to manufacturer's instructions of the Vectastain Universal ABC-AP kit (Vector Laboratories, Burlingame, California, U.S.A), which uses an avidin-biotin complex method and Fuchsin as the chromogen. Phospho-Akt, Ser473 (#9277 L, Cell Signalling, MA. USA) was used at a dilution of 1:50 and incubated with sections for 18 hours at 4°C. Positive (prostate cancer tissue) and negative controls were included in each staining procedure. Samples were assessed as described previously.

## Results

**Figure 1:**
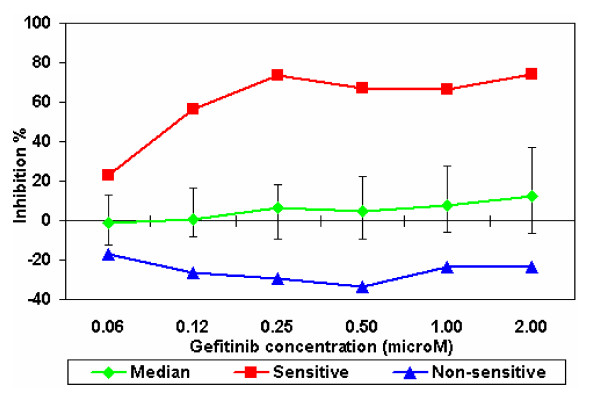
Median effect of gefitinib on tumour-derived cells compared with a sensitive and non-sensitive colorectal tumour. Error bars show 25^th ^and 75^th ^inter-quartile range.

Gefitinib showed low inhibition (Index_SUM _>300) across the range of concentrations tested in the ATP-TCA, with little evidence of increasing inhibition with increasing drug concentration. 7% (6/86) of tumours exhibited considerable inhibition (>50% inhibition at 100% TDC), but most showed a more modest response resulting in a low maximum percentage inhibition (Figure 1). The estimated median IC50 and IC90 value for single agent gefitinib in all tumours tested was 3.98 microM (<0.1–69.9 microM) and 6.45 microM (2.4–125.9 microM) respectively. The median IC50 for individual tumour types tested is shown in Table 2.

**Figure 2:**
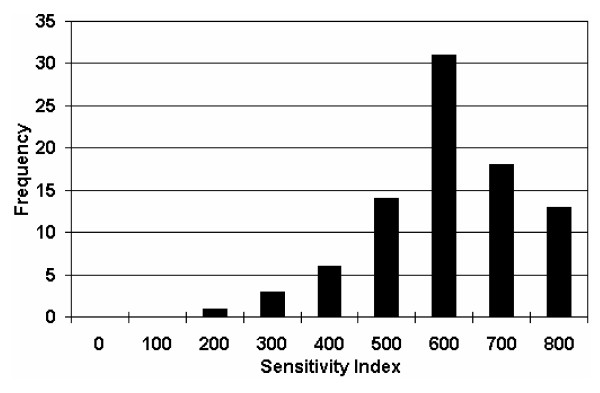
Frequency histogram showing heterogeneity of the Index_SUM _for gefitinib alone in all tumours tested (n = 86).

There was heterogeneity in the degree of inhibition observed when tumours were tested against single agent gefitinib (Figure 2). To compare between tumours, an Index_SUM _of <300 corresponding to 50% inhibition across the range of concentrations tested was used to compare results. On this basis, single agent gefitinib was effective against 5% (4/86) of samples, comprising 1 colorectal tumour, 1 ovarian tumour, 1 uveal melanoma and 1 unknown primary carcinoma. In 88% (76/86) of samples there was sufficient material to test gefitinib in combination with different cytotoxics.

Table 3 shows the median results for single-agent cytotoxics tested compared to results when tested in combination with gefitinib. In samples tested with gefitinib in combination with cisplatin (n = 6) only 33% (2/6) showed increased sensitivity (i.e. a decrease in their Index_SUM_), compared to when cisplatin was used alone. The remaining 67% (4/6) showed increased resistance (i.e. an increase in their Index_SUM_). This compares with gefitinib in combination with oxaliplatin (n = 10) where 90% (9/10) of samples showed an increase in sensitivity with the combination, with 1 sample showing a >50% decrease in the Index_SUM_. When gefitinib was combined with gemcitabine (n = 2), both samples showed an increase in their sensitivity.

**Figure 3:**
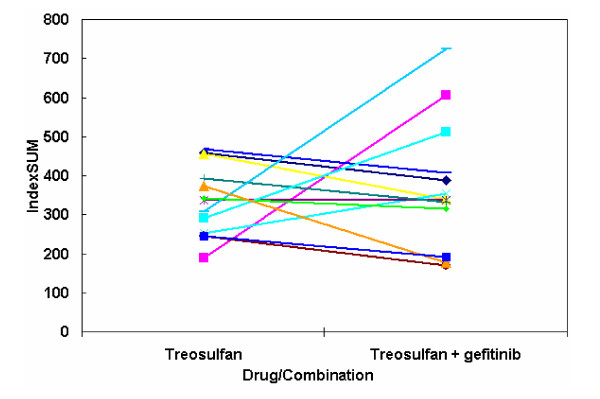
The effect of gefitinib on tumour-derived cells from recurrent ovarian cancer (n = 13). Legend Some tumours show increased sensitivity (lower Index_SUM_), while others show enhanced resistance (higher Index_SUM_)

**Figure 4:**
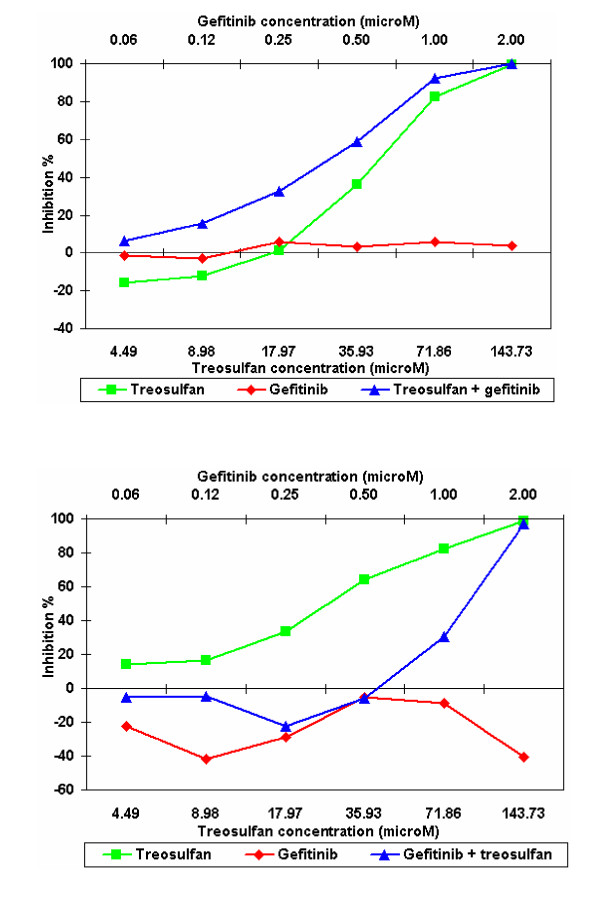
Tumour growth inhibition by gefitinib vs. treosulfan + gefitinib in 2 different tumour types. Legend Figure 4a shows the positive effect of combining treosulfan plus gefitinib in cells derived from a skin melanoma compared with figure 4b in which the combination of treosulfan plus gefitinib has a negative effect in cells derived from an ovarian tumour.

Of the tumours tested with treosulfan + gefitinib, 38% (13/34) were of ovarian origin. Of these, 62% (8/13) showed potentiation, with 1 sample showing a >50% decrease in Index_SUM_. 31% (4/13) showed increased resistance with the combination in comparison with treosulfan alone and 1 sample showed no change (Figure 3). Of the remaining samples tested with gefitinib + treosulfan, 57% (12/21) showed an increase in sensitivity, 38% (8/21) showed an increase in their resistance and one sample showed no change. Figures 4a and 4b show differential effects of gefitinib in combination with treosulfan in cells derived from a skin melanoma sample (Figure 4a) and an ovarian carcinoma sample (Figure 4b). When gefitinib was tested in combination with treosulfan + gemcitabine (n = 24), 54% (13/24) showed an increase in sensitivity, with 3 samples showing a >50% decrease in their Index_SUM _and 46% (11/24) showed an increase in resistance.

In summary, the addition of gefitinib appeared to potentiate the effect of the cytotoxic agent or combination in 59% (45/76) of tumours tested; of these 11% (5/45) had a >50% decrease in their Index_SUM_. In 38% of tumours (29/76), the combination of gefitinib + cytotoxic caused the Index_SUM _to increase thereby increasing resistance. In the remaining 3% (2/76) there was no change observed.

Immunostaining for EGFR was positive in 32% (10/31) of samples comprising of 1 breast carcinomas, 5 colon carcinomas, 1 ovarian carcinoma, 1 sarcoma, 1 skin melanoma and 1 carcinoma of unknown primary site. Immunostaining for pAkt was positive in 81% (25/31) of samples comprising of 3 breast carcinomas, 10 colon carcinomas, 2 oesophageal carcinomas, 2 ovarian carcinomas, 4 skin melanomas, 3 uveal melanomas and 1 carcinoma of unknown primary site. Of the positive samples, 8 were positive for both antibodies (comprising 1 breast carcinoma, 4 colon carcinomas, 1 ovarian carcinoma, 1 skin melanoma and 1 carcinoma of unknown primary site). In 74% (23/31) of samples that were stained for EGFR and pAkt, there was an IC50, IC90 and Index_SUM _value available for comparison. In all cases tested there was no relationship with gefitinib activity and EGFR or pAkt staining.

## Discussion

This is the first study in which a TKI has been successfully tested in the ATP-TCA. ATP-TCA has potential to assist drug development for TKIs and possibly to direct therapy for individual patients. It represents one possible answer to the need for predictive oncology testing of these agents, and could be performed alongside clinical trials to obtain correlation data with outcome in patients treated with gefitinib. However, it is difficult to ascertain whether these were specific or non-specific effects of gefitinib and whether similar outcomes would be seen in the clinical setting. This would need to be determined before using this test for routine screening. Gefitinib showed activity in the assay and even though cytotoxic effects were not expected, in some cases the diminution in ATP levels suggests that these may occur. In general, flat concentration – activity curves were observed which are consistent with a cytostatic rather than a cytotoxic effect. Gefitinib alone showed activity in lung, ovarian and colon carcinomas. These results were consistent with previous findings in cell lines [24].

**Figure 5:**
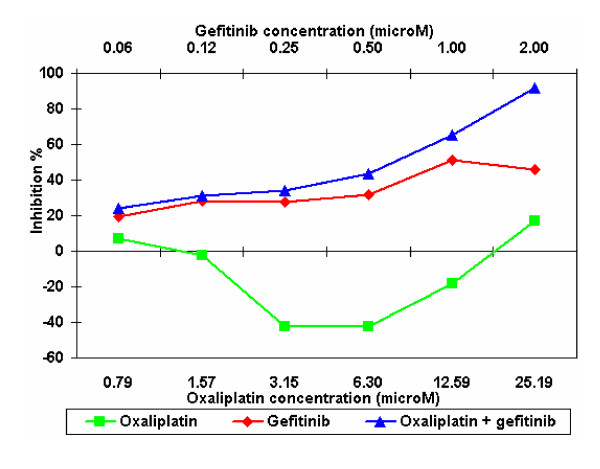
Tumour growth inhibition by gefitinib vs. gefitinib + oxaliplatin in a NSCLC. Legend The combination of oxaliplatin + gefitinib has a positive effect compared with single agent oxaliplatin which is inactive.

When gefitinib was tested in combination with a limited number of cytotoxic drugs, increases and decreases in the activity of the cytotoxic agent were observed. For example, gefitinib in combination with cisplatin caused 67% of samples to have a decrease in the activity of the cytotoxic. This compares with gefitinib in combination with a second platinum-containing agent, oxaliplatin, where 91% of samples showed an increase in the activity of the cytotoxic. However, it should be noted that oxaliplatin was virtually ineffective against the cells tested and this is therefore likely to reflect the effect of the gefitinib alone (Figure 5). Decreased activity of cytotoxic agents when these were combined with gefitinib was seen in 4 samples with cisplatin, 13 with treosulfan, 1 with oxaliplatin and 11 with treosulfan + gemcitabine. This could be detrimental to patients. It is similar to the effect of tamoxifen treatment on the success of breast cancer chemotherapy [25].

Although there was heterogeneity in the response of tumours to single agent gefitinib, there was no relationship between immunostaining for EGFR and gefitinib activity, consistent with other published studies [26]. Sirotnak *et al*. [9] showed that gefitinib caused growth inhibition in human tumour xenografts that was not dependent on high levels of EGFR expression. However, EGFR activation leads to activation of at least three separate second messenger cascades. While the ras/raf pathway may mediate the proliferative effects, survival signals are thought be mediated by the PI3/Akt pathway. As cells have to die in the ATP-TCA to register increased inhibition, sensitivity might be related to the degree of activation of the Akt pathway by other mechanisms. Sensitivity to gefitinib and other non-TKI EGFR inhibitors might therefore be related to pathway activation assessed by detection of pAkt, rather than the levels of EGFR expression. However, this study has not found any such relationship and, when EGFR staining was compared to pAkt staining there was no correlation between EGFR levels to pAkt activity. A similar observation was made by Campiglio *et al*., [27] whose data suggested that neither MAPK nor pAkt were reliable markers of gefitinib activity. It should be noted that many receptors lead to Akt activation and that constitutive activation of the PI3/Akt pathway may be the result of PTEN inactivation.

Of the 4 samples that had an Index_SUM _of <300 and the 6 samples that demonstrated >50% inhibition at 100% TDC when tested with single agent gefitinib, 2 samples (a uveal melanoma and an unknown primary) had material available for immunohistochemical staining with EGFR and pAkt. The uveal melanoma was negative for EGFR and positive for pAkt compared to the unknown primary, which was positive for both EGFR and pAkt. However, there were samples with similar IHC results that did not show sensitivity to gefitinib. As the EGFR (HER1) dimerizes with the other HER molecules and mediates greater activity as a heterodimer, it is likely that the expression of these molecules is also important to the activity of gefitinib [12]. Sensitivity to gefitinib is therefore likely to be the end result of a complex series of interactions within the cell.

## Conclusion

In this study we have found that gefitinib in combination with different cytotoxic agents (cisplatin; gemcitabine; oxaliplatin; treosulfan and treosulfan + gemcitabine) is a double-edged sword: their effect on growth rate may make some tumours more resistant to concomitant cytotoxic chemotherapy, while their effect on cytokine-mediated cell survival (anti-apoptotic) mechanisms may potentiate sensitivity to the same drugs in tumours from other individuals.

## Competing interests

IAC is director of Cantech Ltd.

## Authors' contributions

LAK drafted the manuscript and carried out ATP-TCA assays. FDN, PW, SM, SS and SG also carried out ATP-TCA assays. PJ carried out the immunohistochemical work and IAC conceived the study and participated in its co-ordination.

## Pre-publication history

The pre-publication history for this paper can be accessed here:



## Figures and Tables

**Table 1:** Drug concentrations used in the ATP-TCA

**Drug**	**TDC (microM)**
Cisplatin	10.0
Gemcitabine	40.0
Gefitinib	1.0
Oxaliplatin	12.6
Treosulfan	71.9

**Table 2:** Median IC50 (microM) and Index_SUM _values for single-agent gefitinib for all tumours tested.

**Tumour**	**N**	**IC50 (microM)**	**Index_SUM_**
Breast adenocarcinoma	8	7.27 (6.2–16.9)	607 (500–785)
Colorectal adenocarcinoma	18	3.19 (0.1–52.6)	568 (239–818)
Melanoma – cutaneous (CMEL)	7	2.81 (1.9–29.4)	514 (471–587)
Melanoma – uveal (UMEL)	12	17.10 (0.04–69.9)	595 (187–746)
NSCLC	1	-	396
Squamous cell carcinoma	2	2.83 (2.3–3.4)	462 (454–469)
Oesophageal adenocarcinoma	4	3.58 (2.8–4.4)	602 (456–788)
Ovarian carcinoma	26	3.09 (0.2–23.1)	534 (269–777)
Carcinoma of unknown primary site (UPS)	5	4.76 (0.05–14.3)	612 (258–648)
Sarcoma	2	9.8	609 (588–630)
Sweat gland carcinoma	1	24.67	651

(Negative values of IC50 have been excluded as meaningless. Negative values usually resulted from flat concentration – activity curve).

**Table 3:** Median results for single-agent cytotoxics tested compared with results when tested in combination with gefitinib.

**Drug/Combination**	**N**	**AUC**	**IC90**	**IC50**	**Index_SUM_**	**% showing decrease in Index_SUM _when in combination with gefitinib**
Gefitinib	86	3943 (40–13212)	646 (243–12614)	399 (4–7008)	570 (187–818)	-
Cisplatin	6	7937 (4244–9804)	294 (208–557)	132 (84–309)	434 (382–497)	-
Cisplatin + gefitinib	6	9006 (1731–12057)	225 (159–585)	107 (58–325)	486 (351–588)	33% (2/6)
Gemcitabine	2	7127 (498–13756)	764 (206–1321)	382 (30–734)	452 (288–616)	-
Gemcitabine + gefitinib	2	12605 (9835–15374)	211 (201–220)	52 (17–86)	315 (207–422)	100% (2/2)
Oxaliplatin	10	3488 (874–8884)	833 (317–2267)	463 (129–1259)	559 (379–681)	-
Oxaliplatin + gefitinib	10	5602 (547–12140)	390 (194–775)	217 (60–431)	447 (310–605)	90% (9/10)
Treosulfan	34	13764 (4351–18390)	146 (35–616)	53 (4–342)	353 (65–726)	-
Treosulfan + gefitinib	34	13656 (4251–18658)	153 (31–19892)	60 (4–11051)	338 (58–897)	56% (19/34)
Treosulfan + gemcitabine	24	17576 (8107–19164)	57 (6–200)	13 (3–110)	155 (21–456)	-
Treosulfan + gemcitabine+ gefitinib	24	17281 (11402–19119)	48 (9–206)	9 (4–70)	146 (25–572)	54% (13/24)
